# A clinical algorithm for triaging patients with significant lymphadenopathy in primary health care settings in Sudan

**DOI:** 10.4102/phcfm.v5i1.435

**Published:** 2013-06-21

**Authors:** Eltahir A.G. Khalil, Imad A. El Hag, Kamal E. Elsiddig, Mohamed E.M.O Elsafi, Mona E.E. Elfaki, Ahmed M. Musa, Brima Y. Musa, Ahmed M. Elhassan

**Affiliations:** 1 Department of Clinical Pathology & Immunology, University of Khartoum, Sudan; 2 Riyadh Military Hospital, Saudi Arabia; 3 Faculty of Medicine, University of Khartoum, Sudan; 4 Central Police Hospital, Burri, Khartoum, Sudan

## Abstract

**Background:**

Tuberculosis is a major health problem in developing countries. The distinction between tuberculous lymphadenitis, non-specific lymphadenitis and malignant lymph node enlargement has to be made at primary health care levels using easy, simple and cheap methods.

**Objective:**

To develop a reliable clinical algorithm for primary care settings to triage cases of non-specific, tuberculous and malignant lymphadenopathies.

**Methods:**

Calculation of the odd ratios (OR) of the chosen predictor variables was carried out using logistic regression. The numerical score values of the predictor variables were weighed against their respective OR. The performance of the score was evaluated by the ROC (Receiver Operator Characteristic) curve.

**Results:**

Four predictor variables; Mantoux reading, erythrocytes sedimentation rate (ESR), nocturnal fever and discharging sinuses correlated significantly with TB diagnosis and were included in the reduced model to establish score A. For score B, the reduced model included Mantoux reading, ESR, lymph-node size and lymph-node number as predictor variables for malignant lymph nodes. Score A ranged 0 to 12 and a cut-off point of 6 gave a best sensitivity and specificity of 91% and 90% respectively, whilst score B ranged -3 to 8 and a cut-off point of 3 gave a best sensitivity and specificity of 83% and 76% respectively. The calculated area under the ROC curve was 0.964 (95% CI, 0.949 – 0.980) and -0.856 (95% CI, 0.787 - 0.925) for scores A and B respectively, indicating good performance.

**Conclusion:**

The developed algorithm can efficiently triage cases with tuberculous and malignant lymphadenopathies for treatment or referral to specialised centres for further work-up.

## Abstrait

**Un algorithme clinique pour le triage des patients atteints de lymphadénopathie importante dans les milieux de soins de santé primaires au Soudan**

**Présentation:**

La tuberculose est un problème de santé majeur dans les pays en voie de développement. La distinction entre tuberculose ganglionnaire, lymphadénite non spécifique et l’élargissement malin des ganglions lymphatiques doit être opérée au niveau des centres de soins de santé primaires en adoptant des méthodes simples à appliquer et peu coûteuses.

**Objectif:**

Développer un algorithme clinique pour les centres de soins de santé primaires afin de trier les cas de tuberculose ganglionnaire, de lymphadénite non spécifique et des lymphadénopathies tuberculeuses et malignes.

**Méthodes:**

Le calcul des rapports de cote (RC) des variables prédictives sélectionnées a été réalisé par régression logistique. Les valeurs numériques des notes des variables prédictives ont été pondérées par rapport à leur RC respectif. Les caractéristiques de la note ont été évaluées par la courbe de la fonction d'efficacité du récepteur (courbe ROC).

**Résultats:**

Les quatre variables prédictives (test Mantoux, vitesse de sédimentation érythrocytaire, fièvre nocturne et écoulement des sinus) étaient étroitement corrélées au diagnostique de tuberculose et ont été intégrées dans le modèle réduit pour établir une note A. Pour la note B, le modèle réduit incluait le test de Mantoux, la vitesse de sédimentation érythrocytaire, la taille des ganglions lymphatiques et le nombre de ganglions lymphatiques, ceci constituant les variables prédictives des ganglions lymphatiques malins. La note A était comprise entre 0 et 12 et un seuil de coupure de 6 produisait respectivement une sensibilité et spécificité idéales de 91% et 90%, la note B étant comprise entre -3 et 8, et un seuil de coupure de 3 produisait respectivement une sensibilité et spécificité idéales de 83% et 76%. La surface calculée sous la courbe ROC était de 0,964 (IC de 95%, 0,949 – 0,980) et de - 0,856 (IC de 95%, 0,787 – 0,925) pour les notes A et B respectivement, indiquant une bonne performance.

**Conclusion:**

L'algorithme développé permet de trier efficacement les cas de tuberculoses et de lymphadénopathies malignes en vue d'un traitement ou d'une orientation vers des centres spécialisés pour un traitement conclusif.

## Introduction

### Key focus

Tuberculosis represents a major health problem in developing countries with strained economies and poor health facilities. Tuberculosis (TB) is a contagious disease of poverty that mainly affects young adults in their most productive years. According to the World Health Organization (WHO) there are about 9.4 million new overt TB cases annually and about two million deaths annually. Most of the TB deaths occur in the developing countries of sub-Saharan Africa.^1^ *Mycobacterium tuberculosis* complex affects a number of organs in humans, giving rise to pulmonary and extra-pulmonary disease phenotypes. The human body harbours hundreds of lymph nodes, together with mucosal associated lymphoid tissue and the spleen as part of the immune system. Lymph nodes and lymphoid collections act as filters for disease-causing agents. In response to foreign antigens lymph nodes initiate immune responses. They are also common sites for the development of secondary and primary neoplasms. Lymph nodes are not normally palpable and people become aware of their presence when they become enlarged (> 1.0 cm) or painful. Most of the cases of enlarged lymph nodes, whether localised or generalised, are explainable or self-limiting and are of a reactive and non-specific inflammatory nature. Good examples of these explainable lymphadenopathies are those secondary to pathology in the area of drainage like the pharynx or the lymph node enlargements that are part of clinical syndromes as infectious mononucleosis. In one epidemiological study, only 10% of cases with enlarged lymph nodes were not explainable and needed referral to a specialist, 3.2% required biopsy and a small minority of 1.1% were malignant.^2^

It is of great medical and economic importance that guidelines should exist by which primary health care staff can effectively and efficiently differentiate between cases with sinister conditions like malignancies, treatable conditions like specific infections and cases of self-limiting disease. Tuberculosis is one of the main specific infections that involve lymph nodes. The negative socio-economic impact of TB has been significantly increased by the HIV and AIDS pandemic.^3, 4, 5^

Tuberculous lymphadenitis is the most common type of extra-pulmonary tuberculosis in developing countries where pulmonary tuberculosis is prevalent. The cervical region is the commonest anatomical site affected.^6, 7, 8^ The incidence of TB in Sudan in 2010 was 119 per 100 000, according to a World Bank report published in 2012.^9^

A distinction between tuberculous, primary or secondary lymph node neoplasms and reactive or non-specific adenitis, the commonest cause of lymphadenopathy, has to be made. The main presenting symptoms of tuberculous lymphadenitis are painless lymph node enlargement, nocturnal fever and discharging sinuses. In addition, the Mantoux test is invariably reactive and the erythrocytes sedimentation rate (ESR) is high in the majority of patients.^7, 10^ Current diagnostic procedures of microbiological identification of the causative agent through special stains, culture or molecular techniques in adjunct to tissue and FNA biopsies are either poorly sensitive or costly. Moreover, some of these techniques are time-consuming whilst others require expertise and equipment which are not readily available in developing countries.^11^ On the other hand, empirical initiation of the anti-tuberculous treatment which is commonly practiced in remote areas can lead to over-treatment of cases with reactive lymph nodes. This in turn may lead to the emergence of drug resistance and unjustified added cost or unnecessary delay in the management of cases with neoplastic lymph node diseases. Clinical algorithms for common health problems similar to TB proved to be effective in countries with meagre financial resources.^12, 13, 14, 15^ Clinical algorithms in the form of flow charts to diagnose and treat cases with significant lymphadenopathy in a cost-effective and efficient way are used in developed countries.^16, 17^ These algorithms break down cases with enlarged lymph nodes step by step, using clinical history as well as radiological and other laboratory tests which may not be available in primary health care settings in developing countries.

As far as we are aware there are no guidelines to sort patients with enlarged lymph nodes in developing countries.

### Objectives

This study aimed to develop simple and cheap screening clinical algorithms to triage fewer patients with significant lymph node enlargement for treatment or further investigation in secondary or tertiary centres.

### Research significance

Most of the diagnostic procedures for lymph node enlargement are time-consuming and expensive. Clinical algorithms that can be employed by primary health care staff for triaging patients with significant lymphadenopathy will save patients’ time, reduce cost, improve management and probably save lives in developing countries.

## Ethical consideration

The research protocol was reviewed and approved by the Scientific and Ethics Committees of the Institute of Endemic Diseases, University of Khartoum. Patients attending the lymphadenopathy clinic were looked after by medically qualified personnel. Patients needing advanced treatment were properly directed.

## Methods

### Design

This was a prospective, cross-sectional and observational study that was conducted during a ten year period (1997-2007) at the Lymphadenopathy Clinic, Institute of Endemic Diseases, University of Khartoum and the Central Police Hospital, Burri, Khartoum, Sudan.

### Patients and methods

After informed consent, patients who presented to the clinic with significant lymphadenopathy were enrolled in this study. Patients were asked to sign a consent form following an adequate explanation of the study protocol. Parents or guardians usually signed for their children. Patients were asked to sign a separate form for anonymous HIV testing as per the local requirement of the Ethical Committee of the Institute of Endemic Diseases, University of Khartoum. Lymphadenopathy was considered significant when generalised, involving supraclavicular and/or deep posterior cervical groups, or the affected lymph-node size was ≥ 1 cm at its largest diameter or lasted for ≥ 2 cm weeks. Demographic data, including name, mother's name, age, gender and locality, as well as clinical and laboratory results were recorded in a specially designed case report form (CRF). Clinical history, including duration of the swelling, chronic cough, prolonged nocturnal fever, recent history of sore throat, joint pain and swelling, weight loss and contact with TB cases, was taken and recorded. Lymph-nodes sites, numbers, approximate size (greatest dimension in cm) and the presence of discharging sinuses were recorded. Mantoux tests, complete blood cell counts and ESR and chest X-rays were performed. Anonymous HIV testing was performed using a rapid diagnostic HIV serological technique. Confirmation was carried out at the National Health Laboratory, Khartoum. Reactive patients were referred for further assessment at the National AIDS Control Program. Fine needle Aspiration cytology (FNAC) was performed for all cases and considered the gold standard for the diagnosis of lymph-node enlargement. Surgical biopsies and histopathology were performed on ten patients only when repeated FNAC sampling was insufficient or the cytology results were inconclusive.

PCR using *Mycobacteria tuberculosis* species-specific primers (IS6110) was performed for patients with huge nodes (6 cm - 9 cm) and reactive cytology results. Patients with chronic debilitating illnesses such as diabetes mellitus, chronic renal failure and pulmonary tuberculosis as well as those with a history of immunosuppression (HIV and/or AIDS) were excluded. Patients on immunosuppressive drugs and patients who were involved in other clinical trials were also excluded.

### Procedure

#### Fine needle aspiration cytology (FNAC)

Twenty-gauge needles were used for lymph node aspiration. Slides were air-dried, fixed in absolute methanol, stained with May-Gründwald Geimsa and examined under a 100X oil emersion objective lens. Trained and experienced pathologists examined the lymph nodes’ cytology. The diagnosis of tuberculosis lymphadenitis was based on the presence of certain cytological features that include paucity of cells with a predominance of polymorphs with occasional small lymphocytes on a ground glass background with caseation necrosis and epithelioid granulomas. Patients with tuberculous lymphadenitis were assigned to the following cytomorphological categories: necrotising, granulomatous and necrotising granulomatous.

#### The Mantoux skin test

Five units of PPD in 0.1 ml were injected intra-dermally in the volar aspect of the forearm. As negative control 0.1 ml of sterile phosphate buffered saline (PBS) was injected intra-dermally on the volar aspect of the contra-lateral forearm. The induration was read using the ball-point pen technique after 48 hours to 72 hours, where the mean of the two largest diameters was calculated.

### Analysis

#### Development of a clinical diagnostic score

A two-step clinical scoring system (score A and B) was established using multinomial logistic regression to break down cases of lymphadenopathy into three groups: TB lymphadenitis, reactive or non-specific adenitis and malignancy (metastasis and lymphoma). Score A was applied first to exclude TB cases, after which score B was applied to separate reactive and malignant lymph nodes. The A and B scores were developed in two phases. In the first phase of the model, correlation between predictor variables, e.g. the Mantoux result, ESR and nocturnal fever, and the outcome variable or clinical diagnosis (TB vs. non-TB and reactive vs. malignant) was performed, using the ANOVA variance analysis for continuous variables and Chi square test for dichotomous variables. In the second phase the model was reduced by excluding predictor variables which were not significantly associated (*p* > 0.05) with the outcome variable. Using multinomial logistic regression, *β*-coefficients and ExpB, which is the odd ratio (OR) of the predictor variables, were determined. The numerical score values of the predictor variables were weighed against their respective ORs; a score of 0 for an OR of 0 – 0.9, 1 for an OR of 1 – 1.9, 2 for an OR of 2 – 2.9 and 3 for an OR of 3 and above. A negative score value was given for predictor variables having a significantly (*p* < 0.05) negative *β*-coefficient. The model goodness of fit was evaluated by using Pearson and Deviance statistics. Model fitting information was obtained by the likelihood ratio test. The performance of the score was evaluated by the ROC (Receiver Operator Characteristic) curve. All statistical analyses were performed with an SPSS software programme.

## Results

**FIGURE 1:**
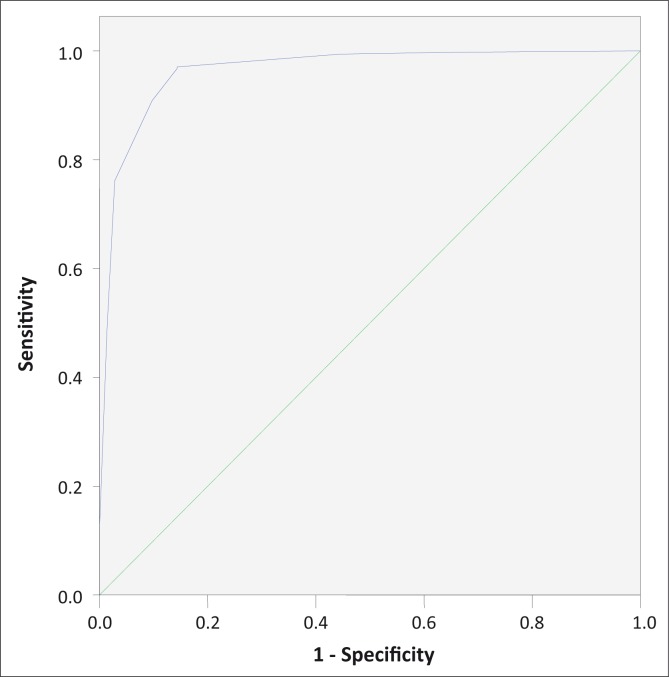
A receiver operating characteristic (ROC) curve for Score A.

Five hundred and fifty-five consecutive and eligible individuals were admitted to this study. The demographic data, FNAC diagnosis and the correlation between the predictor variables (Mantoux induration in mm, ESR value, Hb concentration, nocturnal fever, sinuses, and number and size of lymph nodes) and the various diagnoses as outcome variables are summarised in Tables 1 and 2. One patient (1/555, 0.1%) tested positive for HIV. Samples were later sent for confirmation to the National Health Laboratory, and the patient was referred for counselling and further management at the National AIDS Control Program. Four patients (4/555, 0.7%) with reactive cytology tested positive for *M.tuberculosis* in the PCR test, and these patients were excluded from the analysis. Patients who had undergone surgical biopsy and histopathology were also excluded from the analysis. Four predictor variables, namely Mantoux induration in mm, ESR value, nocturnal fever and discharging sinuses were found to correlate significantly with TB diagnosis and therefore included in the reduced model to establish score A. For score B, the reduced model included the Mantoux reading, ESR, lymph node size and lymph node number as predictor variables. The multinomial logistic regression analysis of the reduced model for score A is shown in Table 3. The Pearson and Deviance statistics (chi square of 8.7 and 8.1 and *p-*value of .85 and .89, respectively based on 14 degrees of freedom) proved the model's goodness of fit. The final model is shown to out-perform the null model in the likelihood ratio test with a *p-*value of less than 0.001 based on 8 degrees of freedom. The derived score, ranging from 0 to 12, is shown in Table 4. The area under the ROC curve (Figure 1) is 0.964 (95% CI, 0.949 – 0.980), indicating a good performance of the score. A cut-off point of 6 gives the best result in terms of sensitivity and specificity (91% and 90%, respectively). A likelihood ratio (LR) of 9.1 indicates that the performance of the clinical algorithm is very good and that there is a good possibility that patients with Tuberculous lymphadenitis will be ruled in. This will give an improved triaging ability over that obtained if positive predictive values are used, which to a large extent is dependent on disease prevalence.

**TABLE 1:** Demographic, clinical and laboratory data: Comparison between various Fine Needle Aspiration Cytology diagnoses using one-way ANOVA variance analysis or Chi-square test.

Diagnosis	Variables	Reactive (*n* = 159)	TB (*n* = 339)	Metastasis (*n* = 28)	Lymphoma (*n* = 29)	*p*-value
Gender	Male	102	171	15	19	0.020
	Female	57	168	13	9
Age	Years (**±** s.d)	24 ± 17	28 ± 15	45 ± 19	31 ± 19	0.001
ESR	Millimetres/hour (**±**s.d.)	41 ± 26	90 ± 27	41 ± 39	85 ± 36	0.001
Mantoux	Millimetres (±s.d.)	10 ± 9	19 ± 6	5 ± 7	0 ± 1	0.001
Hb	Grams/deciliter (±s.d.)	12 ± 2	11 ± 2	13 ± 2	11 ± 3	0.140
L. node	Count (±s.d.)	2 ± 1	2 ± 2	2 ± 1	3 ± 2	0.001
L. node	Size in centimetres (±s.d.)	4 ± 2	5 ± 2	7 ± 6	7 ± 3	0.001
Fever	Frequency	8%	84%	11%	17%	0.001
Sinuses	Frequency	0%	17%	0%	0%	0.001

TB, Tuberculosis; ESR, Erythrocytes sedimentation rate; Hb, Hemoglobin; L. node, Lymph node.

**TABLE 2:** Demographic, clinical and laboratory data: Comparison between TB and non-TB groups using one-way ANOVA variance analysis and Chi-square test.

Diagnosis	Variables	TB	Non-TB	*p*-value
Gender	Male	171	1369	0.004
	Female	168	80
Age	Years (**±** s.d)	28 ± 15	28 ± 19	0.63
ESR	Millimetres/hour (**±**s.d.)	90 ± 28	47 ± 33	0.001
Mantoux	Millimetres (±s.d.)	19 ± 6	8 ± 9	0.001
Hb	Grams/deciliter (±s.d.)	11 ± 2	12 **2**	0.047
L. node	Count (±s.d.)	2.1 ± 1.7	1.7 ± 1.7	0.004
L. node	Size in centimetres (±s.d.)	5.0 ± 2.4	4.7 ± 3.4	0.199
Nocturnal Fever	Frequency	84%	10.70%	0.001
Sinuses	Frequency	17%	0%	0.001

TB, Tuberculosis; ESR, Erythrocytes sedimentation rate; Hb, Hemoglobin; L. node, Lymph node.

**TABLE 3:** Predictors of TB diagnosis based on multinomial logistic regression analysis. The table gives OR, *p*-values and numerical score.

Predictor	Variable (mm)	Multivariate OR	95% CI	*p*-value	Score
Sinus	-	3.2E7	3.2E7 – 3.2E7	< 0.001	3
Nocturnal Fever	-	24.8	12.0 – 51.4	< 0.001	3
Mantoux	< 10	-	-	-	0
	10 – 19	2.5	1.2 – 5.5	0.02	2
	≥ 20	45.9	17.5 – 120.1	< 0.001	3
ESR	< 60	-	**-**	**-**	0
	≥ 60	14.6	6.9 – 30.6	< 0.001	3

TB, Tuberculosis; OR, Odd ratio; ESR, Erythrocytes sedimentation rate.

**TABLE 4:** Score A to identify TB cases with sensitivity, specificity, positive and negative likelihood ratios.

Score	Sensitivity	Specificity	LK+	LK-
0	1.00	0.00	1	0
2	1.00	0.37	1.6	0
3	0.99	0.56	2.25	0.02
4	0.97	0.86	6.9	0.03
5	0.97	0.86	6.9	0.03
6	0.91	0.9	9.1	0.1
7	0.76	0.97	25	0.2
8	0.76	0.97	25	0.2
9	0.49	0.99	49	0.5
11	0.12	1	Absolute	0.9
12	0.1	1	Absolute	0.9

TB, Tuberculosis; LK, Likelihood.

**FIGURE 2:**
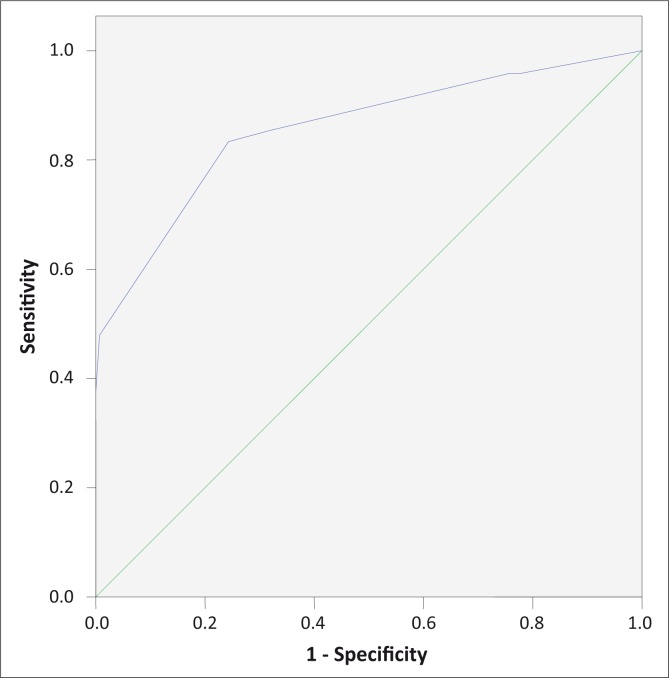
A receiver operating characteristic (ROC) curve for Score B.

The multinomial logistic regression analysis of the reduced model for score B is shown in Table 5. The model is found to fit the data adequately, as shown by Pearson and Deviance goodness of fit statistics (chi square of 15.9 and 15.3 and *p*-value of .07 and .08 based on 9 degrees of freedom). The final model is shown to outperform the null model in the likelihood ratio test with a *p*-value of less than 0.001 based on 4 degrees of freedom. The derived score, ranging from -3 to 8, is shown in Table 6. The area under the ROC curve (Figure 2) of -0.856 (95% CI, 0.787 - 0.925) indicated a satisfactory performance of the score. A cut-off point of 3 gives the best result in terms of sensitivity and specificity, 83% and 76% respectively. An LR of 3.5 makes this algorithm at least three times more predictive for ruling in the disease.

**TABLE 5:** Predictor of malignant lymph node diagnosis based on multinomial logistic regression with OR, *p*-values and numerical score.

Predictor	Variable (mm)	Multivariate OR	95% CI	*p*-value	Score
Mantoux	< 10	-	-	-	0
	≥ 10	0.11	0.04 – 0.36	< 0.001	-3
ESR	< 80	-			0
	≥ 80	9.9	3.6 – 27.6	< 0.001	3
L. node count	≤ 2	-	-	-	0
	>2	2.9	0.9 – 9.3	0.066	2
L. node size	≤ 4	-	-	-	0
	>4	7.3	2.9 – 18.7	< 0.001	3

TB, Tuberculosis; ESR**, E**rythrocytes sedimentation rate; L. node, Lymph node; OR, Odd ration.

**TABLE 6:** Score B to differentiate between malignant and reactive lymph-nodes. The table gives sensitivity, specificity, positive and negative likelihood ratios.

Score	Sensitivity	Specificity	LK+	LK-
3	1	0	1	0
-1	0.95	0.22	1.2	0.2
0	0.95	0.24	1.3	0.2
2	0.85	0.68	2.7	0.2
3	0.83	0.76	3.5	0.2
5	0.48	0.99	48	0.5
6	0.37	0.99	37	0.6
7	0.13	1	Absolute	0.9
8	0.1	1	Absolute	0.9

TB, Tuberculosis; LK, Likelihood.

## Discussion

Lymphadenopathy is a worrying clinical symptom that is mostly painless and benign. The infectious nature of tuberculous lymphadenitis is well elucidated and *M. tuberculosis* was reported as the common causative organism in developing countries.^9^ In remote areas, a large number of patients with reactive non-specific lymphadenopathy, lymphomas or secondary malignant deposit are empirically treated with anti-tuberculosis drugs, with disastrous consequences. In Sudan and many other African countries primary health care facilities are very poor and unable to treat the simplest of cases properly. Unnecessary costs, long delays and probably a loss of lives are incurred in the management of patients with lymphadenopathy in remote areas of these countries. Except for the FNAC technique diagnostic procedures, such as microbiological culture and PCR, are lengthy and require costly equipment and well-trained staff. The under-funded and under-staffed health centres concentrate on other medical conditions that are believed to be more important than painless lumps in the neck. The chronicity and the non-infectious nature of some lymphadenopathies put them lower down on the priority list of medical conditions in developing countries. Empirical anti-tuberculous treatment is widely practiced by primary health care staff who have very little knowledge and training.

Clinical algorithms have been used in clinical practice for some time now and they are intended to triage patients that require special procedures or care at more specialised centres. The use of clinical algorithms greatly improves patient management in countries with meagre resources. Clinical algorithms for the triaging patients in developing countries should be simple, cheap and easy to implement and should need minimal training. The use of such clinical algorithms greatly improves patient management in countries with meagre health resources.^10, 11, 12, 13, 14^ We foresee that the algorithms presented here will be able to sort pathological from non-pathological lymphadenopathies initially. This will then hasten the diagnosis of cases of tuberculous and malignant lymphadenopathies. The guidelines should therefore improve the skills of primary health care staff to pick up cases that are eligible for further advanced management. The algorithms are also intended to reduce costs, avoid unnecessary diagnostic delays and relieve patients with reactive non-specific lymphadenopathy from the misery of lengthy empirical drug treatments. The algorithms should also be able to pick up patients with malignant lymphadenopathies at an early stage of the disease for timely treatment and therefore the possibility of improved survival rates.

High hopes are invested in this score, because it is simple and uses sets of symptoms, signs and simple, cheap diagnostic tests and can easily be introduced by staff at primary healthcare centres. Score A will differentiate tuberculous from non-tuberculous (reactive/malignant) nodes with high sensitivities and specificities that are ≥ 90%, giving the medical staff a good chance to introduce anti-tuberculous drugs with a high degree of certainty. Using score B on the rest of the patients will allow the staff to identify patients with reactive or non-specific lymphadenopathy that do not need treatment or referral with reasonably high sensitivities and specificities of more than 75%. A very small number of patients suspected of having malignant lymph node enlargement will filter to the next step where they should be referred for further sophisticated diagnostic procedures at secondary or tertiary levels.

This study clearly showed that multiple lymph nodes with nodes sizes ≥ 2 cm correlated well with an FNAC diagnosis of pathological lymphadenopathy. Likewise it was shown that patients with reactive or non-specific lymph node enlargement are generally well with few nodes, a non-reactive Mantoux test (induration = 00 mm) and a low ESR. It was also noted that nocturnal fever and sinuses were strongly associated with an FNAC diagnosis of tuberculous lymphadenopathy. General ill health, lymphadenopathy involving more than one group of lymph nodes with nodes sizes ≥ 2, non-reactive mantoux (00 mm induration) and a high ESR correlated well with an FNAC diagnosis of lymphoma. This initial screening gives medical history and clinical examination the added value of triaging patients even before applying any form of full clinical scoring system.

### Recommendations

Our results were obtained from a prospective and a hospital-based study that was carried out at specialised centres and institutions that are well equipped with skilled and highly trained staff. This situation is obviously different from that of primary health care centres in under-privileged and remote areas. Therefore our recommendation is that the derived algorithms be validated in a larger study involving staff at a primary health centre who have undergone a reasonable period of training.

## Conclusion

In conclusion we are of the opinion that a good medical history and clinical examination can initially sort patients into normal and those with pathological lymphadenopathies. The derived algorithm can efficiently sort tuberculous, reactive and malignant lymphadenopathies, reduce cost and prevent detrimental delays in management. However, further testing in primary health care settings with adequate training for staff is strongly recommended.
